# Identification of Molecular Subtypes and Prognostic Features for Triple-Negative Breast Cancer Based on Golgi Apparatus-Related Gene Signature

**DOI:** 10.32604/or.2025.061757

**Published:** 2025-07-18

**Authors:** Zhun Yu, Jie Wang, Guoping Xu

**Affiliations:** 1 Department of Breast, International Peace Maternity and Child Health Hospital, School of Medicine, Shanghai Jiao Tong University, Shanghai, 200030, China; 2 Shanghai Key Laboratory of Embryo Original Diseases, Shanghai, 200030, China

**Keywords:** Triple-negative breast cancer (TNBC), Golgi apparatus (GA), prognostic model, molecular subtypes, drug sensitivity

## Abstract

**Objectives:** Triple-negative breast cancer (TNBC) presents a major treatment challenge due to its aggressive behavior. The dysfunction of the Golgi apparatus (GA) contributes to the development of various cancers. This study aimed to utilize GA-related genes (GARGs) to forecast the prognosis and immune profile of TNBC. **Methods:** The data were downloaded from The Cancer Genome Atlas (TCGA) database, including 175 TNBC and 99 healthy samples. The differentially expressed GARGs (DEGARGs) were analyzed using the TCGA biolinks package. The patients with TNBC were classified into two clusters utilizing the ConsensusClusterPlus package according to prognosis-related DEGARGs, followed by comparing the differences in prognosis and immune infiltration between the two clusters. Next, LASSO and stepwise Cox regression were applied to establish a GARGs signature to forecast the TNBC prognosis. The association of the GARGs signature with immune infiltrates and drug sensitivity was further explored. **Results:** In total, 430 DEGARGs were identified between TNBC and healthy samples, among which 20 were related to TNBC prognosis. Two GARG-related molecular clusters associated with different survival times and immune heterogeneity were identified. A risk model for TNBC was established based on six GARGs, and the high-risk (HR) group exhibited a poor prognosis. The HR group demonstrated a distinctly high M2 macrophage infiltration and low M1 macrophage infiltration, which contributed to an immunosuppressive tumor microenvironment and thus led to poor prognosis of the HR group. Immune dysfunction scores and programmed cell death ligand 1 (PD-L1) expression were substantially elevated in the HR group. The HR group showed increased sensitivity to anticancer drugs, such as cisplatin. **Conclusion:** Our findings suggest that GARGs are involved in the pathogenesis of TNBC and provide new insights into prognostic prediction. The identified clusters and GARGs signatures have the potential to guide individualized therapy.

**Highlights**

1. Two clusters were identified based on Golgi apparatus-related genes (GARGs).
2. A prognostic model was constructed by six GARGs.
3. The high-risk group had a poor prognosis and a high abundance of M2 macrophages.
4. Low-risk patients may benefit more from immunotherapy.
5. The high-risk group was more sensitive to anticancer drugs such as cisplatin.

## 1 Introduction

Triple-negative breast cancer (TNBC) accounts for 15%–20% of all breast cancers and is characterized by a lack of specific molecular biomarkers, including estrogen receptor, progesterone receptor, and human epidermal growth factor receptor 2 (HER2) [1]. Due to the lack of molecular targets, treatments mainly include appropriate surgery and chemotherapy [2]. TNBC is highly aggressive and metastatic, with distant metastasis significantly shortening patient survival time [3]. Therefore, understanding its underlying mechanisms and exploring therapeutic targets are necessary.

The Golgi apparatus (GA) is the central organelle of the secretory pathway that is responsible for the progression of soluble cargo (proteins and lipids) synthesized by the endoplasmic reticulum and then distributed to the correct cellular compartment [4]. Considering that GA dynamics are finely regulated, abnormalities in its structure and function affect cellular lipid and protein homeostasis [5]. As a central hub for intracellular transport, synthesis, and modification, GA participates in key biological processes, including protein synthesis, modification, and localization [6].

Increasing evidence reveals that GA dysfunction is involved in the development of various diseases, including cancer [7]. Several Golgi apparatus-related genes (GARGs) are linked to cancer prognosis. For instance, Jiang et al. [8] developed a GARGs-based risk model to forecast clinical prognosis and guide immunotherapy in patients with lung adenocarcinoma. Sun et al. [9] also used the GARGs signature to predict prognosis and therapeutic response of hepatocellular carcinoma. Liu et al. [10] found that the GARGs signature could enable the prediction of the postoperative progression-free interval of patients with papillary thyroid cancer. In breast cancer, modifications in GA function and organization are found to contribute to cancer development [11]. Changes in N-glycan remodeling and metabolism in the GA contribute to epithelial-mesenchymal transition and metastasis in patients with breast cancer patients [12]. Abnormal expression or mutations in specific GARGs have been observed in breast cancer, providing potential insights into prognostic evaluation [13]. In TNBC, protein kinase D3 (PKD3) (localized in the GA) is highly expressed in cancer samples and can provide a molecular link between the GA and endolysosomes to enhance the transduction of proliferation signals, like mechanistic target of rapamycin complex 1-ribosomal protein S6 kinase 1 (mTORC1-S6K1) [14]. Functional disruption of the GA protein ADP-ribosylation factor 1 (ARF1) enhances the sensitivity of TNBC cells to both Actinomycin D and Vinblastine, leading to reduced cell proliferation and migration and increased apoptosis [15]. However, the relationship between GARGs and TNBC prognosis has not yet been confirmed, and is a novel direction for exploration.

With the development of bioinformatics, many prognostic models have been developed to predict TNBC prognosis and immune activity [16–18]. In this study, based on data from public databases, a series of bioinformatics analyses were utilized to establish a GARG-related model for prognosis prediction and immune response in TNBC. The findings of this study may improve TNBC prognosis prediction accuracy and contribute to the development of personalized treatment strategies, ultimately enhancing the clinical management and therapeutic outcomes for TNBC patients.

## 2 Materials and Methods

### 2.1 Datasets Collection and Screening

RNA-seq data of patients with TNBC and corresponding clinical information (including age, sex, race, and primary diagnosis), as well as survival information (overall survival status and overall survival time), were retrieved from The Cancer Genome Atlas (TCGA, https://portal.gdc.cancer.gov/, accessed on 18 July 2023), including 180 cancer and 99 healthy samples. Among these, three patients were alive but had missing overall survival times. To investigate TNBC, the annotation information of 180 TCGA samples that had been identified in the study by Lehmann et al. [2] was downloaded, including sample number (IDs) and subtype information. Paraffin-embedded samples and those lacking clinical information were removed, and 99 healthy individuals and 175 patients with TNBC were selected for analysis.

The TCGA biolinks package [19] was used to normalize the downloaded raw data and remove low-quality genes. Gene expression profiles of GSE58812 (containing 107 TNBC samples) [20] and GSE21653 (including 266 breast cancer samples) [21] were acquired from Gene Expression Omnibus (GEO, https://www.ncbi.nlm.nih.gov/geo/, accessed on 21 July 2023), which were utilized as validation cohorts.

### 2.2 Differential Analysis of GARGs

In total, 1643 GARGs in the Golgi pathway (GOCC_GOLGI_APPARATUS) were obtained from the gene set enrichment analysis (GSEA) database (http://www.gsea-msigdb.org/gsea/index.jsp, accessed on 19 July 2023) [22]. The ssGSEA scores of GARGs in TNBC and healthy control samples were calculated and compared using the ssGSEA method. Higher ssGSEA scores indicated increased activity of the Golgi apparatus pathway in TNBC. Moreover, differential expression analysis of GARGs between TNBC and healthy control samples was performed using the TCGA biolinks package [19]. The calculated *p*-value was then corrected for multiple testing using the Benjamini-Hochberg method. The thresholds of differentially expressed GARGs (DEGARGs) were: false discovery rate (FDR) < 0.05, and |log_2_ fold change (FC)| > 1.

### 2.3 Screening of Prognosis-Related DEGARGs

To assess the prognostic value of these DEGARGs, the R language 4.3 survival package (v 2.41-1, https://github.com/therneau/survival, accessed on 19 July 2023) was used for univariate Cox regression analysis. Genes related to overall survival were selected with *p*-value < 0.05.

### 2.4 Cluster Analysis

Based on the prognosis-related DEGARGs, the ConsensusClusterPlus package [23] was used to perform cluster analysis on 175 TNBC samples to obtain the appropriate number of subtypes.

### 2.5 Survival Analysis

The Kaplan-Meier (KM) curve analysis was conducted using the survminer package (https://rpkgs.datanovia.com/survminer/news/index.html, accessed on 20 July 2023) in R 4.3 to observe the difference in prognosis of patients in different clusters.

### 2.6 Cellular Mutation Analysis

Cellular mutations refer to changes in the genomic DNA sequence, and the analysis of mutation status helps identify driver genes that play key roles in tumor development. In this study, R 4.3 maftools package [24] was used to conduct somatic variant analysis, screen genes, and identify GARGs with significant variation in different clusters.

### 2.7 Correlation Analysis of Molecular Clusters and Immune Infiltration Characteristics

The cell-type identification by estimating relative subsets of RNA transcripts (CIBERSORT) [25] and Estimation of STromal and Immune cells in MAlignant Tumor tissues using Expression data (ESTIMATE) [26] algorithms were used to predict the immune infiltration status of the tumors. For CIBERSORT analysis, the relative cell abundance in each sample was estimated, and the proportion of each immune cell in the samples was obtained. In addition, ESTIMATE was employed to calculate the stromal, immune, and ESTIMATE scores to determine the state of the tumor microenvironment.

### 2.8 Establishment and Validation of the Prognostic GARGs Signature

To better predict patients’ prognosis, a risk model was established utilizing least absolute shrinkage and selection operator (LASSO) Cox regression analysis. First, the glmnet package [27] was applied for cross-validation to select the best penalty parameters for the LASSO model. Second, the Survminer package was employed for stepwise Cox regression analysis, and the Akaike Information Criterion (AIC) was calculated. The optimal combination was determined according to the smallest AIC value. Consequently, the key genes were selected to construct the risk score (RS) model, named the GARGs signature. The formula for constructing RS was as follows: RS = β1X1 + β2X2 + … + βnXn (β and X represent the regression coefficient and gene expression level, respectively). Training and external validation sets (GSE58812 and GSE21653) were utilized to assess the effectiveness of the RS model. Briefly, the samples in each dataset were categorized into low-risk (LR) and high-risk (HR) groups, and prognostic differences between the two risk groups were observed using the KM method in the survival package (v 2.41-1). To examine the performance of this GARGs signature in predicting the 2-, 4-, and 5-year overall survival probabilities, was evaluated using receiver operating characteristic (ROC) analysis was conducted using timeROC (version 0.4) [28]. Additionally, the classic prediction analysis of microarray 50 (PAM50) gene signature [29] was downloaded and subjected to ROC analysis to compare the robustness of the GARGs signature.

### 2.9 Independent Prognostic Factor Prediction

After collecting RS and clinical features (age, race, TNBC type4, and TNM stage), univariate and multivariate Cox analyses were conducted to screen independent prognostic factors.

### 2.10 Nomogram Construction

Based on the independent prognostic factors, we built a nomogram model using the rms package (version 5.1-2) [30] to predict the probability of patients’ survival. The validity of the nomogram was verified using a calibration curve. In addition, the differences in RS among the different subtypes or TNBC types were analyzed.

### 2.11 Evaluation of the Prognostic Value of Genes in the RS Model

To further confirm the effect of the genes in the model on prognosis, survival analysis was performed for each gene. First, the surv_cutpoint function in the Survminer package was utilized to compute the optimal cutoff of gene expression levels, dividing the samples into low- and high-expression groups. Thereafter, KM survival curves were analyzed for both groups.

### 2.12 GESA Calculated the Pathway Enrichment between Different Risk Groups

To explore the kyoto encyclopedia of genes and genomes (KEGG) pathways related to the risk groups, the GSEA database was used. The expression data of samples from different risk groups were entered into the GSEA data, and three key indicators (enrichment score, normalized enrichment score, and *p*-value) were calculated to identify pathways. Significant pathways were selected with a *p*-value < 0.05.

### 2.13 Relationship Analysis of RS and Immune Cells

As previously described, CIBERSORT [25] and ESTIMATE [26] were used to analyze the correlation between the LR and HR groups.

### 2.14 Immunotherapy Response and Drug Sensitivity Prediction

Tumor immune dysfunction and exclusion (TIDE) [31] has been applied to assess the degree of tumor immune escape and predict the response to immunotherapy. In addition, the drug sensitivity data were obtained from the Genomics of Drug Sensitivity in Cancer (GDSC) database (https://www.cancerrxgene.org/, accessed on 20 July 2023), and the drug response of each sample was analyzed. Differences in the drug sensitivity of the samples in the two risk groups were compared using the Wilcoxon test.

## 3 Results

### 3.1 Screening of Prognosis-Related DEGARGs in Normal and Cancer Samples

**Figure 1:**
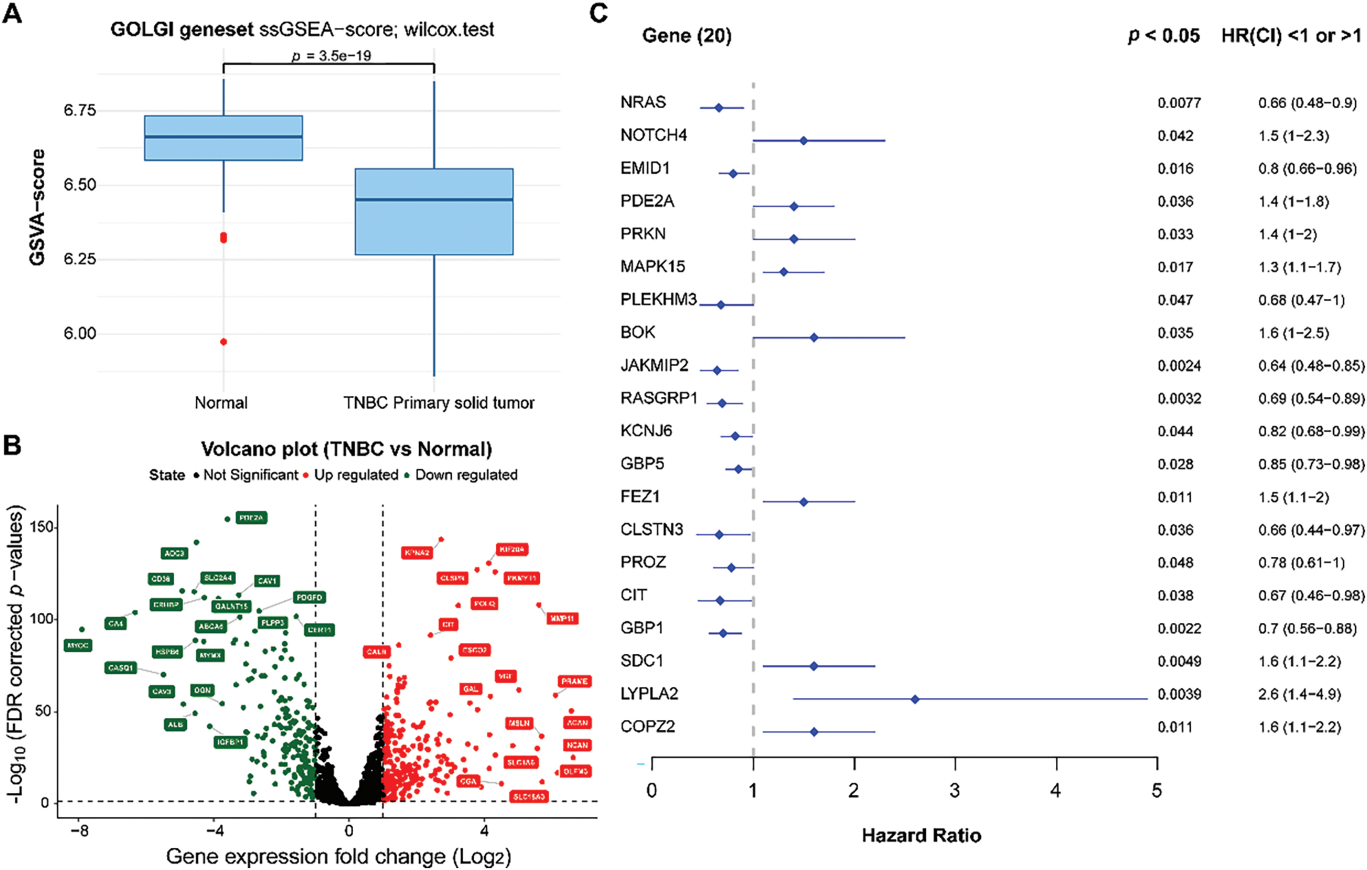
Differential expression and identification of prognosis-related genes. **(A)**: Comparison of Golgi apparatus values between normal and TNBC samples using ssGSEA. **(B)**: Volcano plot of DEGARGs between normal and TNBC. Red and green nodes represent the upregulated and downregulated genes, respectively. **(C)**: Selection of prognosis-related genes using univariate Cox regression analysis

Based on ssGSEA analysis, we observed that TNBC patients had lower ssGSEA scores of GARGs (Fig. 1A), indicating that the activity of the Golgi apparatus pathway was decreased in TNBC. A total of 430 DEGARGs were identified between normal and TNBC samples. DEGARGs are presented using a volcano plot (Fig. 1B). The most significant up-regulated DEGARGs (red nodes) and down-regulated (green nodes) were visualized in this plot. Among DEGARGs, 20 genes were closely associated with prognosis (Fig. 1C). Thereinto, notch receptor 4 (NOTCH4), phosphodiesterase 2A (PDE2A), Parkin RBR E3 Ubiquitin Protein Ligase (PRKN), mitogen-activated protein kinase 15 (MAPK15), BCL2 family apoptosis regulator BOK (BOK), fasciculation and elongation protein zeta 1 (FEZ1), syndecan 1 (SDC1), lysophospholipase 2 (LYPLA2), and coatomer protein complex, subunit zeta 2 (COPZ2) were risk factors (HR > 1, *p* < 0.05), and the remaining DEGARGs are protective factors.

### 3.2 Classification of GARGs-Related Molecular Clusters

**Figure 2:**
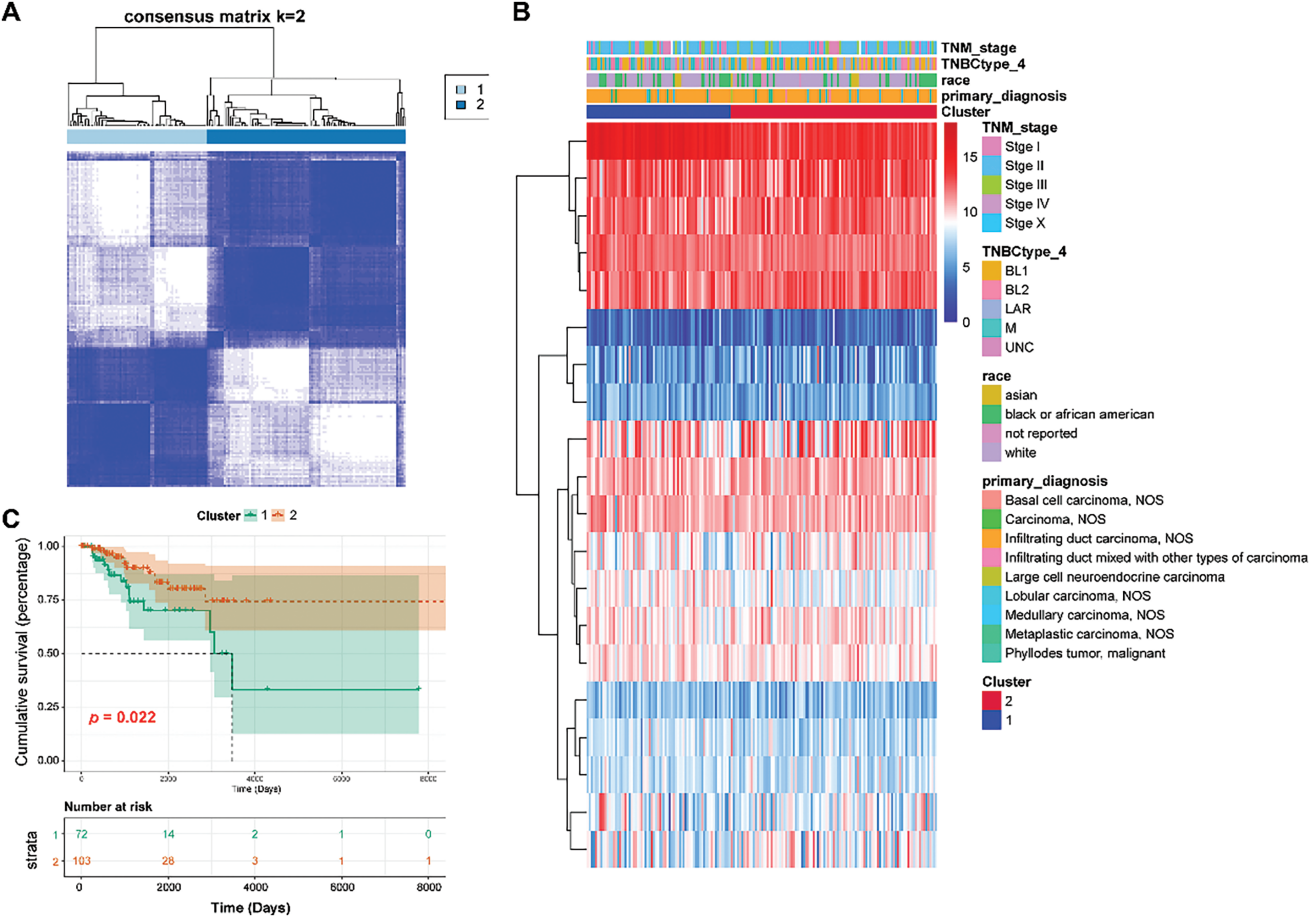
Classification of TNBC patients into two clusters. **(A)**: Two clusters were obtained using the Consensus ClusterPlus analysis. **(B)**: Heat map showing the relationship between clinical features and two clusters. **(C)**: Survival analysis of Clusters 1 and 2 using the KM curve

Cluster analysis was performed on 175 TNBC samples from the TCGA dataset according to 20 prognostic genes. By gradually increasing the clustering variable k from 2 to 6, group clustering was determined to be optimal when k = 2, and two clusters (clusters 1 and 2) were obtained (Fig. 2A). Differences in common clinical indicators (such as TNM stage and race) between the two clusters are demonstrated using a heatmap (Fig. 2B). Moreover, survival analysis showed that patients in Cluster 1 had observably adverse survival times compared to those in Cluster 2 (*p* = 0.022, Fig. 2C).

### 3.3 Mutation Information in Two TNBC Clusters

**Figure 3:**
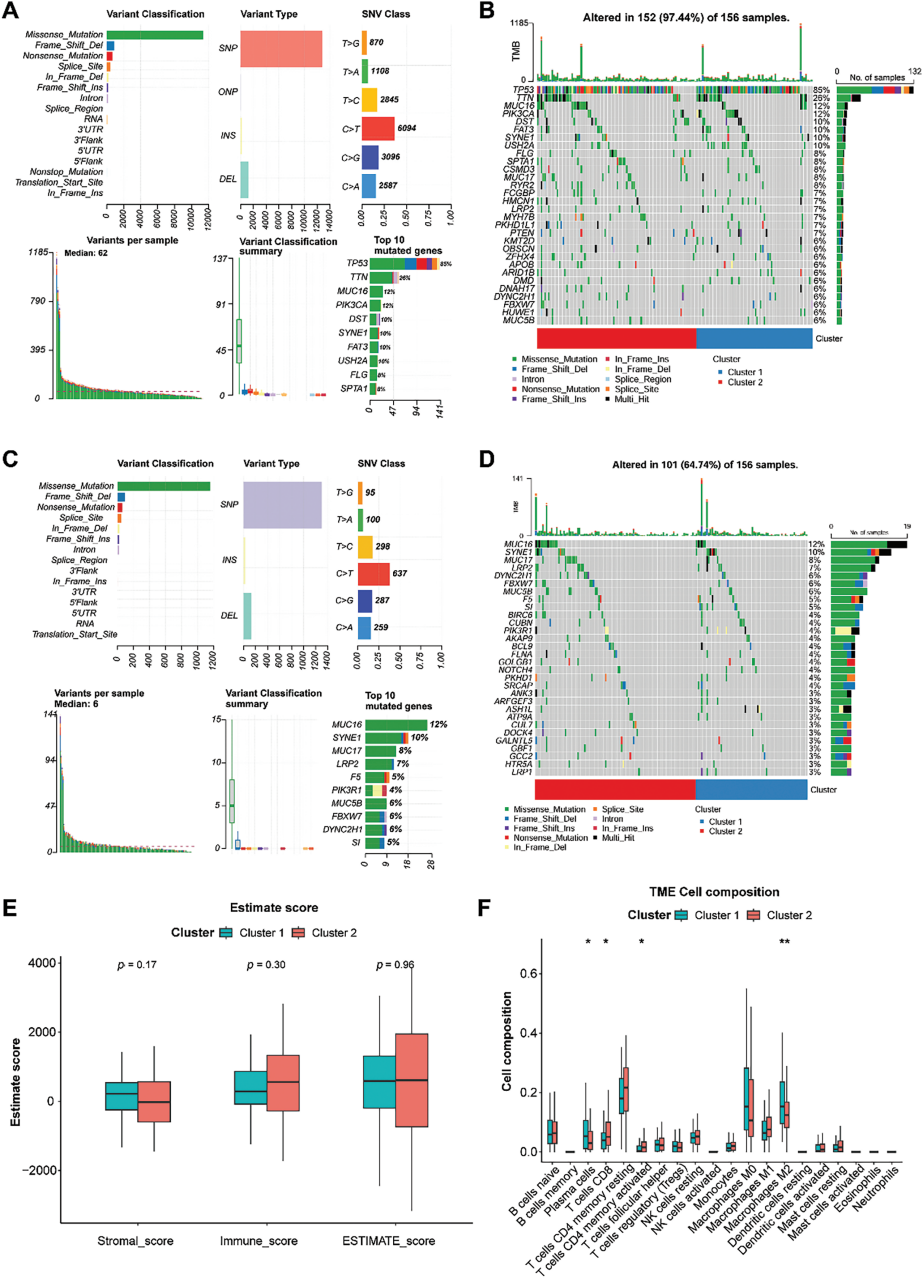
Analysis of somatic mutation and immune characteristics between the two clusters. **(A)**: Mutation information for all genes in TNBC samples. **(B)**: Genes with high mutation frequencies in the two clusters. **(C)**: Mutation information for GARGs in TNBC samples. **(D)**: Genes with high mutation frequencies in the two clusters. **(E)**: Comparison of stromal, immune, and ESTIMATE scores between clusters 1 and 2. **(F)**: Differential analysis of 20 immune cell types in clusters 1 and 2. *p < 0.05; **p < 0.01

A total of 163 TNBC samples with somatic mutations were downloaded from the TCGA dataset. Statistical analysis revealed that missense mutations in all genes occurred predominantly in TNBC, with the majority belonging to the single nucleotide polymorphism (SNP) type (Fig. 3A). Among all the genes, tumor protein 53 (TP53) had the highest mutation frequency (85%), followed by Titin (TTN) (26%). Genes with high mutation frequencies in the two clusters are displayed in Fig. 3B. These mutations, particularly in TP53, might have significant implications for TNBC progression and prognosis, as TP53 is a critical tumor suppressor gene that participates in DNA repair, cell cycle regulation, and apoptosis [32]. Additionally, missense mutations in GARGs occurred predominantly in TNBC, with most of them classified as SNP (Fig. 3C). Among the GARGs, mucin 16 (MUC16) had the highest mutation frequency (12%) in both clusters, followed by SYNE1 (10%). MUC16 is known to play a role in cell division and metastasis [33], and its mutation is common in TNBC patients [34]. Therefore, MUC16 mutation could potentially enhance the metastatic potential of TNBC cells, further complicating treatment strategies. GARGs with high mutation frequencies in the two clusters are displayed in Fig. 3D.

### 3.4 Immune Infiltration of Two TNBC Clusters

The stromal, immune, and ESTIMATE scores did not differ significantly between the two groups (Fig. 3E). Compared with cluster 1, cluster 2 showed higher infiltration of CD8 T cells and activated memory CD4 T cells and lower infiltration of plasma cells and M2 macrophages (all *p* < 0.05, Fig. 3F). However, a significant difference was not observed in other immune cells between the two clusters (Fig. 3F).

### 3.5 Establishment and Validation of a Prognostic GARGs Signature

**Figure 4:**
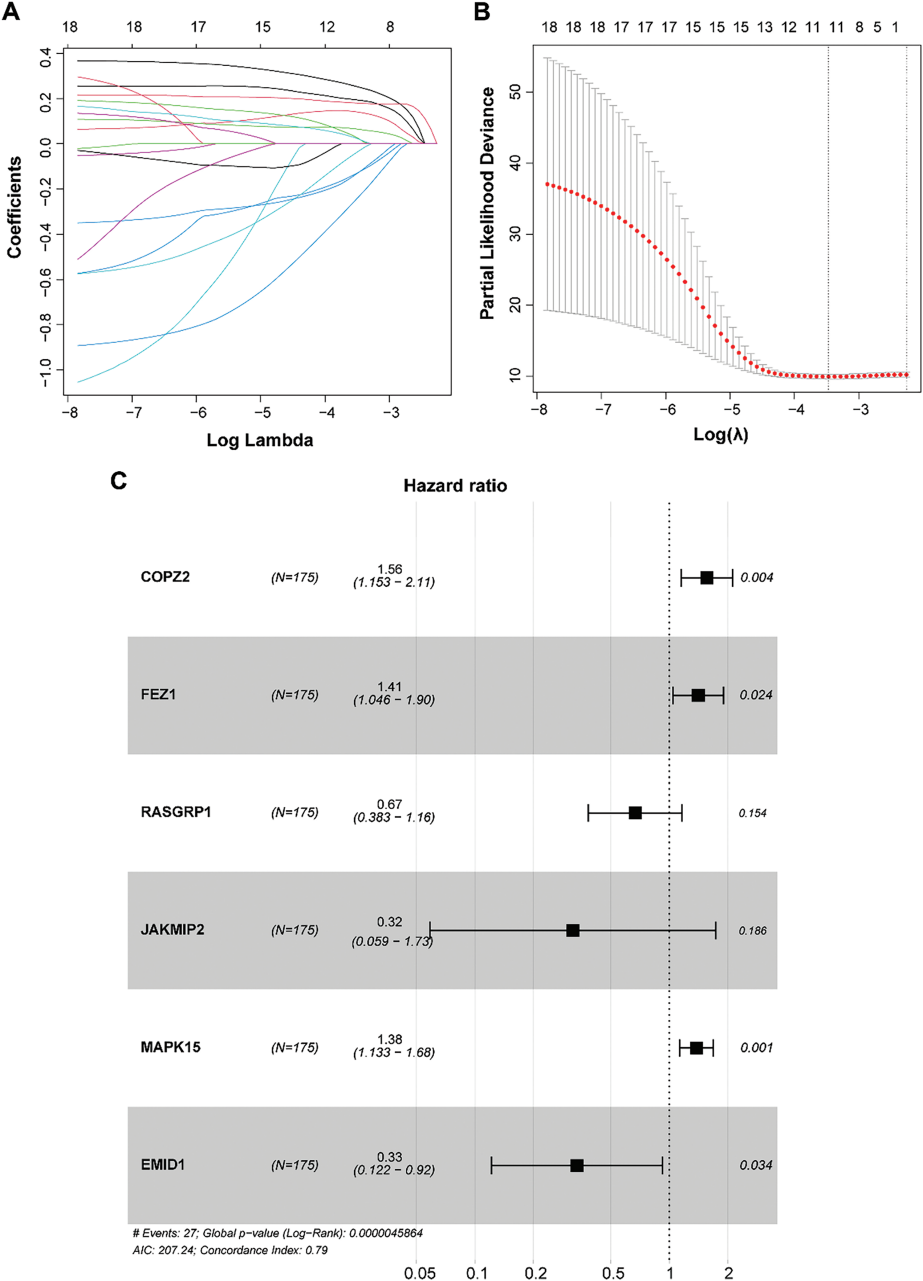
Construction of a prognostic model based on GARGs signature. **(A)**: LASSO coefficient curves of the 17 GARGs. **(B)**: Optimal penalty parameters of the LASSO model selected by cross validation. **(C)**: Stepwise Cox regression was used to identify the key genes for prognostic model construction

To develop and validate the prognostic GARGs signature, the expression of 20 prognosis-related DEGARGs was observed in the GSE58812 validation set. However, two genes, citron Rho-interacting serine/threonine kinase (CIT) and PRKN, were not recorded. Hence, 18 GARGs were selected for further prognostic modeling. LASSO regression analysis identified 17 characteristic genes (Fig. 4A,B). To further simplify the signatures and obtain core genes, a stepwise regression analysis was carried out. Based on the smallest AIC value, six key GARGs, COPZ2, FEZ1, RAS guanyl-releasing protein 1 (RASGRP1), janus kinase and microtubule interacting protein 2 (JAKMIP2), MAPK15, and EMI domain containing 1 (EMID1), were screened to build a prognostic model, containing COPZ2, FEZ1, RASGRP1, JAKMIP2, MAPK15, and EMID1 (Fig. 4C). RS = 0.4449 × ExpCOPZ2 + 0.3423 × ExpFEZ1 − 0.4041 × ExpRASGRP1 − 1.1410 × ExpJAKMIP2 + 0.3231 × ExpMAPK15 − 1.0941 × ExpEMID1.

**Figure 5:**
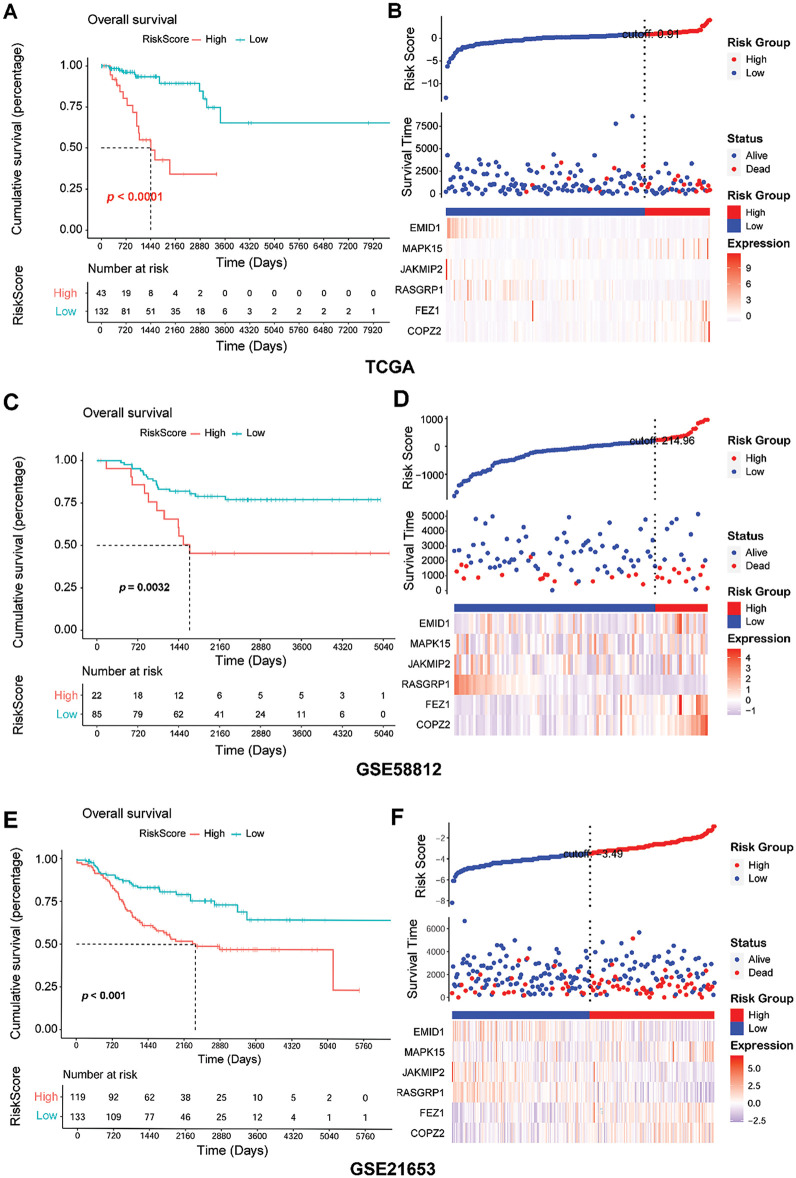
Assessment of predictive performance of GARGs signature in internal and external cohorts. **(A)**: Kaplan–Meier analysis of overall survival between the LR and HR groups in the TCGA. **(B)**: Distribution of RS and survival states in the TCGA. **(C)**: KM analysis of the overall survival between the LR and HR groups in GSE58812. **(D)**: Distribution of RS and survival states in GSE58812. **(E)**: KM analysis of overall survival between the LR and HR groups in GSE21653. **(F)**: Distribution of the RS and survival states in GSE21653. LR, low-risk; HR, high-risk

To understand the predictive performance of the prognostic GARGs signature, a series of bioinformatic analyses were performed. In the training (TCGA) and validation (GSE58812 and GSE21653) datasets, the patients were categorized into LR and HR groups based on the RS threshold. In TCGA, the patients in the HR group had a significantly shorter overall survival (*p* < 0.0001; Fig. 5A). In the HR group, a high proportion of dead samples were observed; the expression levels of MAPK15, FEZ1, and COPZ2 increased, whereas those of EMID1, JAKMIP2, and RASGRP1 decreased (Fig. 5B). Comparable results were seen in the GSE58812 (Fig. 5C,D) and GSE21653 (Fig. 5E,F) cohorts. Briefly, patients in the HR group had an unfavorable prognosis (Fig. 5C,E). Higher RS values were associated with more dead samples, and changes in the expression of the six genes were consistent with those observed in the training set (Fig. 5D,F). Furthermore, the prognostic GARGs signature had a high predictive power for 2-, 4-, and 5-year survival in TNBC patients with an area under the curve (AUC) of 0.788, 0.815, and 0.863 in the TCGA training set (Supplementary Fig. S1A), and had moderate predictive power, with an AUC of 0.748, 0.611, and 0.633 in the GSE58812 validation set (Supplementary Fig. S1B) and 0.523, 0.619, and 0.623 in the GSE21653 validation set (Supplementary Fig. S1C). Notably, the GSE58812 and GSE21653 datasets were derived from different platforms and patient cohorts, which might introduce potential batch effects, technical variations, and heterogeneity between the datasets. These differences could impact the predictive power of the GARGs signature across diverse patient populations. To validate the robustness and stability of the GARGs signature, ROC analysis was performed to analyze the predictive power of the PAM50 signature for the three datasets. The PAM50 gene signature also had a high predictive power for 2-, 4-, and 5-year survival in TNBC patients with an AUC of 0.894, 0.960, and 0.969 in the TCGA training set (Supplementary Fig. S1D), but had a low predictive power with an AUC of 0.771, 0.539, and 0.573 in the GSE58812 validation set (Supplementary Fig. S1E), and 0.365, 0.453, and 0.434 in the GSE21653 validation set (Supplementary Fig. S1F). Overall, these findings suggested that the predictive prognostic ability of the GARGs signature was reliable.

### 3.6 RS as an Independent Prognostic Factor

**Figure 6:**
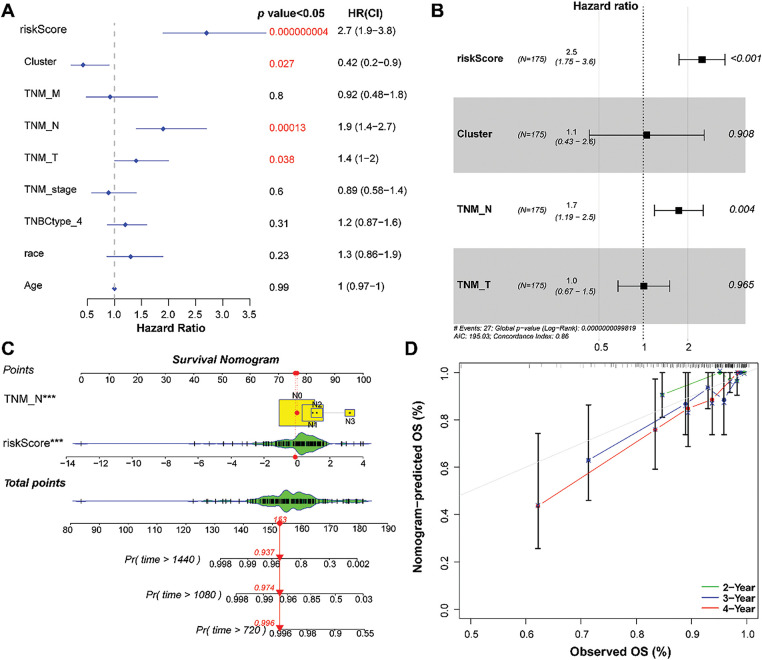
Establishment of a nomogram based on independent prognostic factors. **(A)** and **(B)**: Univariate and multivariate Cox regression analyses of the RS and clinical parameters. **(C)**: Establishment of a nomogram to predict the 2-, 3-, and 4-year survival of patients with TNBC. **(D)**: Calibration curve showing consistency between the predicted and actual survival. ***p < 0.001

Common clinical information (cluster, TNM stage, TNBC type, race, and age) and the RS of the patients were collected. Univariate and multivariate Cox analyses were performed to screen for independent prognostic factors. The results revealed that RS and N staging were identified as independent prognostic factors (Fig. 6A,B).

### 3.7 Nomogram Construction and Evaluation

Based on RS and N staging, a nomogram was developed to forecast overall survival (Fig. 6C). Calibration curves showed that the 2-, 3-, and 4-year overall survival rates predicted by the nomogram agreed with the observed results (Fig. 6D).

### 3.8 Prognosis Analysis of Genes in the RS Model

**Figure 7:**
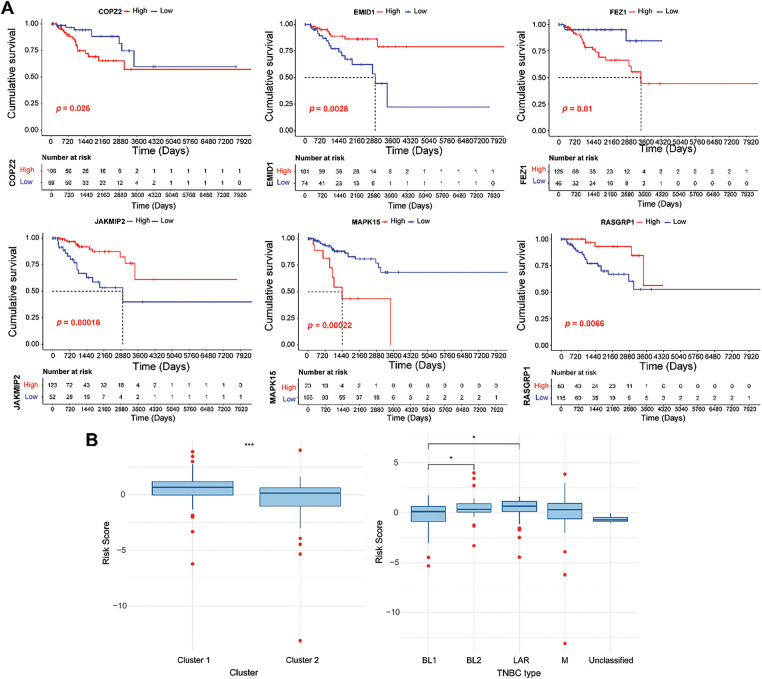
Survival analysis of six genes in the GARGs signature. **(A)**: KM curves of the six GARGs with low and high expression levels. **(B)**: Differences in RS between different molecular clusters and TNBC types. *p < 0.05; ***p < 0.001

Based on the expression of six model genes, patients were classified into low- and high-expression groups, respectively. As shown in Fig. 7A, high COPZ2, FEZ1, and MAPK15 expression and low EMID1, JAKMIP2, and RASGRP1 expression were related to poor cumulative survival. Furthermore, the RS values in different clusters and the four TNBC types (LAR, M, BL1, and BL2) were compared. The results indicated that the RS value of cluster 2 was lower than cluster 1, which might explain the better prognosis of cluster 2 (Fig. 7B). As clusters 1 and 2 had different mutation and immune infiltration characteristics, the differences in RS values between clusters 1 and 2 might be driven by a combination of mutations and immune infiltration.

### 3.9 GSEA Results

**Figure 8:**
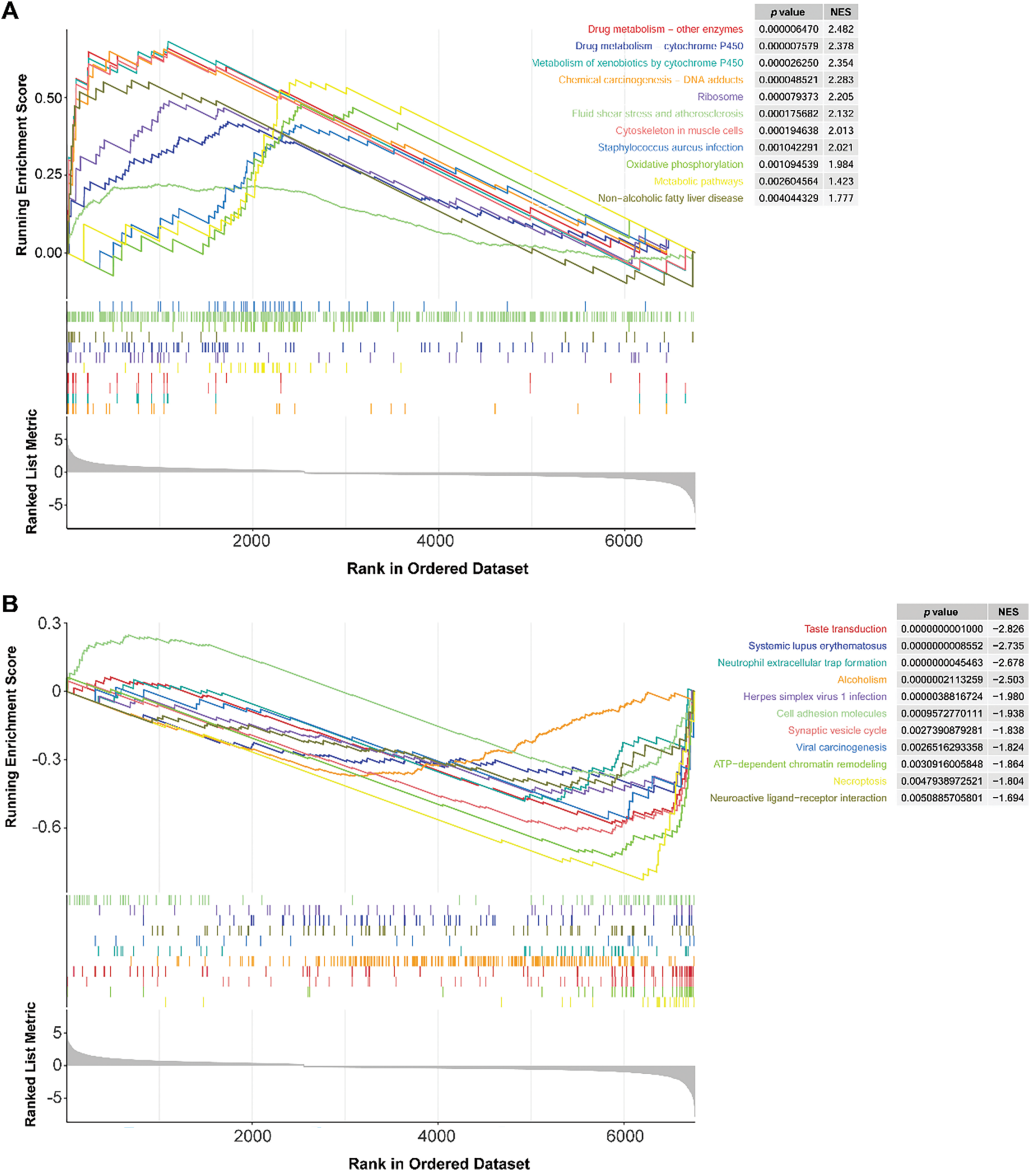
GSEA results of the LR and HR groups. **(A)**: KEGG pathways were mainly enriched in the HR group. **(B)**: KEGG pathways are mainly involved in the LR group. LR, low-risk; HR, high-risk

The GSEA method identified 22 differential pathways between the two risk groups. Of these, 11 KEGG pathways were enriched in the HR group, such as drug metabolism-cytochrome P450 and metabolism of xenobiotics by cytochrome P450 (Fig. 8A). Cytochrome P450 activity has been shown to modulate the fate of anticancer agents and drug resistance [35]. These pathways might play a potential role in drug resistance and metabolism in TNBC. In contrast, 11 KEGG pathways were involved in the LR group, such as cell adhesion molecules (Fig. 8B). Cell adhesion molecules are found to be related to metastasis and prognosis of TNBC [36,37]. These data indicated that these differential pathways might be the underlying molecular mechanisms that distinguish the two groups of TNBC.

### 3.10 LR and HR Groups Presented Different Immune Profiles

**Figure 9:**
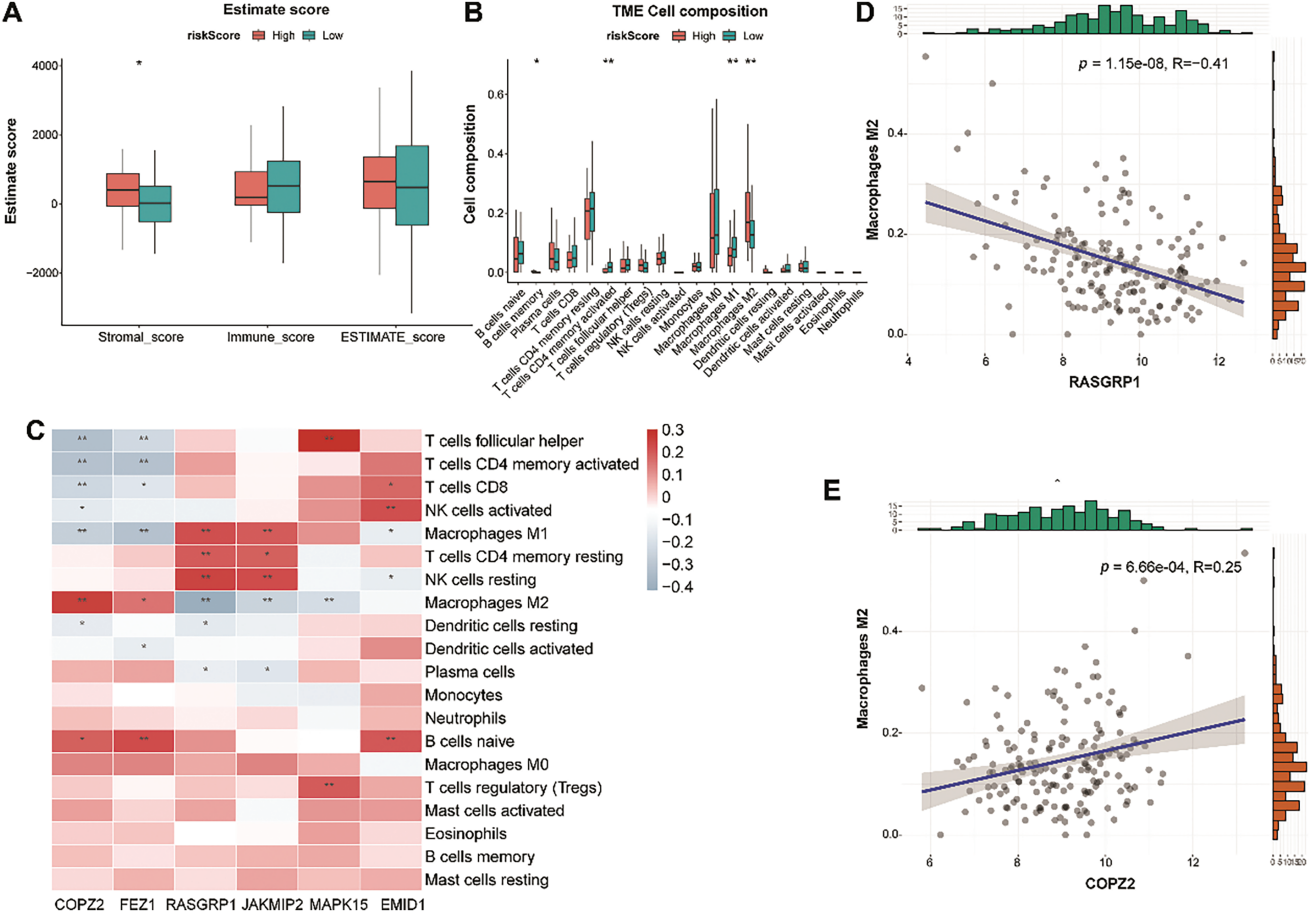
Tumor immune microenvironment analysis between the LR and HR groups. **(A)**: Differences in stromal, immune, and ESTIMATE scores between the two risk groups. **(B)**: Violin plot showing the abundance of 22 immune cells in the LR and HR groups. **(C)**: Relationship between six genes and immune cells. **(D)**: The negative correlation between RASGRP1 and M2 macrophages. **(E)**: The positive correlation between COPZ2 and M2 macrophages. LR, low-risk; HR, high-risk. *p < 0.05; **p < 0.01

To explore the immune infiltration status of the LR and HR groups, we used CIBERSORT to detect the levels of 20 immune cell types. The HR group had a higher stromal value, with high infiltration of M2 macrophages and memory B cells and low infiltration of activated memory CD4 T cells and M1 macrophages (Fig. 9A,B). The heat map revealed the relationship between model genes and immune cells (Fig. 9C). Among these correlations, the most negative and positive correlations were M2 macrophages-RASGRP1, and M2 macrophages-COPZ2, respectively (Fig. 9D,E). Given the finding that M2 macrophage infiltration-related genes have shown the ability to predict immunotherapy in TNBC [38], our data suggest that model genes may affect immunotherapy outcomes of TNBC by affecting M2 macrophage infiltration.

### 3.11 Immunotherapy Response and Drug Sensitivity Analysis

**Figure 10:**
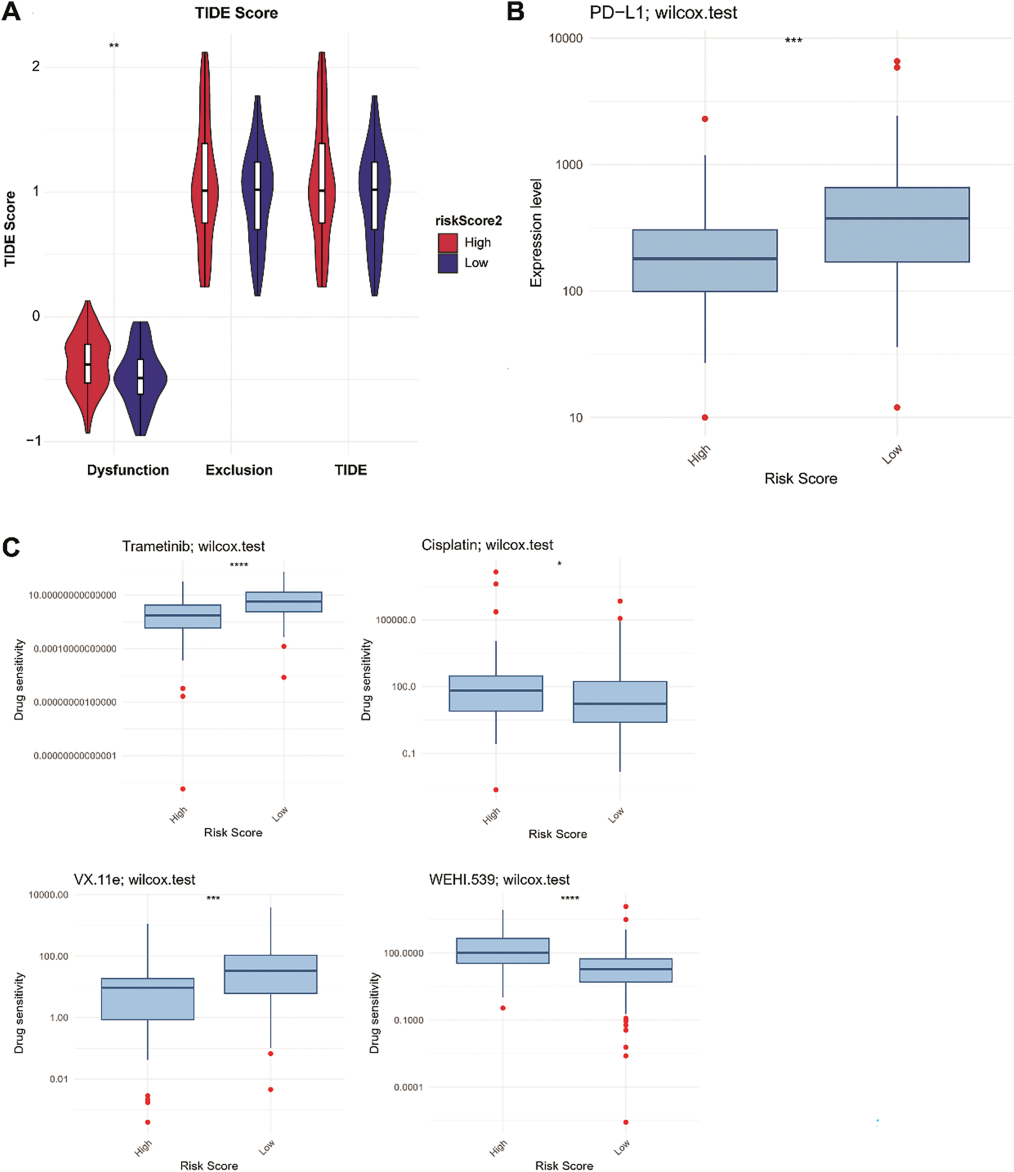
Proportions of immunotherapy response **(A)**, PD-L1 expression **(B)**, and drug sensitivity **(C)** between the LR and HR groups. LR, low-risk; HR, high-risk. *p < 0.05; **p < 0.01; ***p < 0.001; ****p < 0.0001

TIDE analysis indicated that HR patients had significantly higher immune dysfunction scores than LR patients (*p* < 0.01, Fig. 10A), indicating that HR patients might experience a greater degree of immune suppression, which could impair the effectiveness of immunotherapy. Moreover, programmed cell death ligand 1 (PD-L1) expression was higher in the LR group than in the HR group (*p* < 0.001; Fig. 10B). Based on the drug sensitivity values, we observed that the HR group (N = 43) was more sensitive to cisplatin and WEHI.539, indicating that patients in the HR group might benefit from chemotherapy strategies based on cisplatin and WEHI.539. In contrast, the LR group (N = 122) was more sensitive to trametinib and VX-11e (*p* < 0.05; Fig. 10C). Trametinib is a mitogen-activated protein kinase kinase (MEK) inhibitor and can inhibit the growth of TNBC cells [39]. VX-11e is an extracellular signal-related kinases 1 and 2 (ERK1/2) inhibitor, and ERK1/2 signaling plays a role in regulating the migration of TNBC cells [40]. Targeted therapies inhibiting the MEK and ERK1/2 pathways may be more effective in patients in the LR group.

## 4 Discussion

TNBC usually has a poor prognosis due to the lack of a wide range of therapeutic agents and its molecular heterogeneity; thus, it is considered a challenging topic in breast cancer research [41]. The lack of targeted treatment options, combined with the aggressive nature and high potential for metastasis, further complicates the management of TNBC, highlighting the need for novel therapeutic strategies [42]. Although genome sequencing analysis has identified four subtypes of TNBC and improved our understanding of its molecular heterogeneity, these subtypes have not been used in clinical practice [43]. Therefore, further understanding of the molecular characteristics of TNBC could provide new ideas for targeted cancer therapy.

Most previous studies have revealed the mechanism of TNBC tumorigenesis from the perspective of cell function, with limited exploration of the cell structure [44,45]. The function of GA is altered during oncogenic transformation, and this modification may promote cancer cell invasion and metastasis [46]. GA plays a vital role in breast cancer development, including tumor cell proliferation, metastasis, and drug resistance [47–49]. Hence, we identified two GARGs-related clusters with different survival rates and immune cell types in patients with TNBC. Moreover, we developed a GARGs-based prognostic model, providing a new perspective on the prognostic value of GARGs in TNBC.

Consensus clustering, an unsupervised clustering method, recognizes different molecular subtypes based on gene expression clustering [23]. This study identified two GARGs-related clusters with distinct differences in prognosis and immune infiltration. In brief, compared to cluster 2, cluster 1 had a worse prognosis, accompanied by a high infiltration of tumor immune cells, including M2 macrophages. M2 macrophages play a protumorigenic role in the immune microenvironment, modulate tumor cell metastasis and proliferation, and are strongly associated with an aggressive cellular phenotype and poor prognosis [50]. This finding is in line with the results of this study. Because the heterogeneity of TNBC can result in distinct phenotypes and fluctuating clinical outcomes, identifying tumor subtypes and tailoring treatment to individual patients with different phenotypes and prognoses can enhance outcomes [51]. Therefore, exploring GARGs-related clusters could pave the way for improving risk stratification and developing individual treatment strategies for TNBC.

This study developed a prognostic risk model using six core GARGs: COPZ2, FEZ1, RASGRP1, JAKMIP2, MAPK15, and EMID1. COPZ2 encodes a protein that is a subunit of the outer membrane protein complex and functions as a vesicle carrier in the secretory pathway [52]. COPZ2 is confirmed to be linked to the prognosis of bladder and thyroid cancer [53,54]. FEZ1 is frequently altered in human cancers, and its expression affects cell growth by regulating mitosis, which in turn inhibits tumor formation *in vivo* [55]. Meanwhile, the mortality rate of lung cancer patients with high FEZ1 expression has declined [56]. However, we found that FEZ1 overexpression may lead to an adverse prognosis, which is inconsistent with previous reports. It is possible that the complexity and heterogeneity of TNBC affected the results, and validation in cellular or human samples is required. RASGRP1 is localized to the GA and controls T cell development and homeostasis by activating the ERK/MAPK cascade [57]. RASGRP1 has a dual regulatory role in tumor growth and the acute inflammatory response, and TNBC patients with higher expression of RASGRP1 have a better clinical prognosis [58,59], which was also confirmed in our study. JAKMIP2-encoded proteins are components of the Golgi matrix that function as structural scaffolds for the Golgi and negatively regulate the functioning of secretory cargoes [60]. JAKMIP2 is associated with bone metastasis in prostate cancer and is a potential target for improving patient prognosis [61]. MAPK15 mediates the activation of autophagy through starvation, and may be a novel target for small molecule drugs with therapeutic effects in chronic myeloid leukemia [62]. Decreased MAPK15 expression was observed in breast tumors, and loss of MAPK15 was related to breast cancer [63]. EMID1 is associated with immune infiltration levels in lung adenocarcinoma, and overexpression of this gene leads to a favorable prognosis for cancer patients, indicating that EMID1 is an immune-related prognostic marker for lung adenocarcinoma [64]. In this study, all GARGs exhibited significant prognostic value for TNBC, suggesting that these GARGs may serve as prognostic biomarkers for TNBC. In addition, our established GARGs signature exhibited a good ability to predict prognosis in both training and external validation datasets. Moreover, we compared the predictive power of GARGs signature and PAM50 signature in predicting survival, with results showing similar predictive performance. The PAM50 signature, which stratifies breast cancer patients into subtypes based on gene expression patterns, has been extensively validated and remains one of the most widely used prognostic tools in clinical settings [65,66]. Additionally, a previous study has also constructed a GARG-derived risk signature based on 10 GARGs, apolipoprotein A5 (APOA5), ChaC glutathione specific gamma-glutamylcyclotransferase 1 (CHAC1), EMID1, Golgi SNAP receptor complex member 2 (GOSR2), regulator of G protein signaling 20 (RGS20), rabphilin 3A (RPH3A), sarcoglycan epsilon (SGCE), t-complex 1 (TCP1), transmembrane protein 167A (TMEM167A), and zDHHC palmitoyltransferase 15 (ZDHHC15), with 3- and 5-year AUC values of 0.776 and 0.756, respectively [67]. These values were lower than those of our signature (2-, 4-, and 5-year survival AUC values were 0.788, 0.815, and 0.863). Taken together, these findings reveal that our constructed GARGs signature is not only valuable in predicting TNBC prognosis, but also helps tailor personalized treatment by offering insights into the prognosis of patients with different risks.

Furthermore, RS was related to immune cell infiltration. In particular, the degree of macrophage infiltration differed markedly between the LR and HR groups. Macrophages are immune cells that play key roles in both adaptive and innate immunity [68]. They can be polarized under different stimulus conditions to form different phenotypes, including the M1 and M2 types. The infiltration level of the M2 subtype is higher than that of the M1 subtype in TNBC and is associated with a worse prognosis [69]. M1 macrophages exert antitumor activity, whereas M2 macrophages have oncogenic functions [70]. Notably, we observed high and low infiltration of M2 and M1 macrophages in the HR group, respectively, which explains the poor survival of patients in the HR group. Additionally, immune checkpoint genes are central to avoiding self-reactivity and offer promising potential as predictors of immunotherapy effectiveness [71]. PD-L1 expression is often related to a good immunotherapy response [72]. We observed the high PD-L1 expression in the LR group. TIDE analysis also revealed that the immune dysfunction scores were substantially higher in HR patients than in LR patients. Overall, we conclude that patients with LR may benefit more from immunotherapy. Furthermore, patients in the HR group benefited from drugs such as cisplatin. Clinical and experimental studies have demonstrated that cisplatin exerts anti-tumor effects by inducing pyroptosis, thereby improving the pathological complete response rate in patients with TNBC [73]. Moreover, the addition of cisplatin to standard chemotherapy improved the clinical prognosis of locally advanced TNBC patients [74]. Notably, MAPK15 has been found to enhance cisplatin toxicity in various cancer cells and may be a potential therapeutic target [75,76], whereas the interactions between other model genes and cisplatin sensitivity are yet to be fully clarified. Therefore, the risk model constructed can guide clinicians in selecting appropriate drugs for TNBC treatment. However, due to the heterogeneity of TNBC, identifying the cell types that are more sensitive to cisplatin is necessary.

Our study has the following strengths: two molecular subtypes of TNBC related to GARGs were identified for the first time, and corresponding prognostic models were constructed. Moreover, the effects of GARGs on immune cell infiltration and drug responses in TNBC have been observed. Several limitations are presented in this study. First, the sample size and clinical information obtained from public databases were limited, and all samples were retrospective in nature. Thus, the stability of molecular clusters must be confirmed in large prospective cohorts. Second, the expression and role of signature GARGs have not been validated in clinical TNBC samples or through laboratory experiments, and their regulatory mechanisms in TNBC remain experimentally unexplored. Further studies including clinical samples, at least two cell lines *in vitro* and/or 6 animals per group *in vivo* as well as more experiments, such as real-time PCR, western blotting, immunohistochemistry, knockdown or overexpression assays should be performed to clarify how changes in Golgi function influence processes such as cancer cell proliferation, metastasis, or response to therapy in TNBC. Third, the clinical applicability of the GARGs signature for TNBC has not been validated in clinical cohorts. More prospective clinical trials are necessary for real-world validation of the clinical utility of GARGs signature in risk stratification, predicting drug sensitivity, and guiding immunotherapy for TNBC by collecting clinical data on patient outcomes, drug sensitivities, and immune responses. In addition, a close relationship exists between the GARGs signature and immune infiltration; however, their complex interactions need to be thoroughly examined. Further studies should be performed to explore the mechanistic underpinnings of Golgi dysfunction in tumor progression and immune modulation in TNBC, providing new insight into the translational potential of these findings.

## 5 Conclusion

Overall, this study identified two GARG-related molecular clusters with different clinical outcomes and immune infiltration characteristics. We also developed a predictive signature based on six GARGs (COPZ2, FEZ1, RASGRP1, JAKMIP2, MAPK15, and EMID1). Multiple pathways, such as drug metabolism-cytochrome P450 and cell adhesion molecules, might be the underlying molecular mechanisms that distinguish the different risks of TNBC. Moreover, the model genes were linked to multiple immune cell infiltrations, especially M2 macrophage infiltration. Furthermore, patients with LR may benefit more from immunotherapy, and patients with HR benefited from drugs such as cisplatin. The six-gene signatures provided in this study are promising biomarkers that can provide new insights into the individualized treatment of TNBC.

## Supplementary Materials





## Data Availability

The data supporting the findings of this study are available from the corresponding author upon request.

## Supplementary Materials

The supplementary material is available online at https://www.techscience.com/doi/10.32604/or.2025.061757/s1.

## Abbreviations

TNBC: Triple-negative breast cancer
GA: Golgi apparatus
GARGs: Golgi apparatus-related genes
DEGARGs: Differentially expressed GARGs
TCGA: The Cancer Genome Atlas
GEO: Gene Expression Omnibus
FDR: False discovery rate
FC: Fold change
HR: High-risk
PAM50: Prediction analysis of microarray 50
PD-L1: Programmed cell death ligand 1
HER2: Human epidermal growth factor receptor 2
PKD3: Protein kinase D3
ARF1: ADP-ribosylation factor 1
GSEA: Gene set enrichment analysis
CIBERSORT: Cell-type identification by estimating relative subsets of RNA transcripts
ESTIMATE: Estimation of STromal and Immune cells in MAlignant Tumor tissues using Expression data
LASSO: Least absolute shrinkage and selection operator
LR: Low-risk
KM: Kaplan-Meier
RS: Risk score
ROC: Receiver operating characteristic
AUC: Area under the curve
PAM50: Prediction analysis of microarray 50
KEGG: Kyoto encyclopedia of genes and genomes
TIDE: Tumor immune dysfunction and exclusion
GDSC: Genomics of Drug Sensitivity in Cancer
mTORC1-S6K1: Mechanistic target of rapamycin complex 1-ribosomal protein S6 kinase 1
AIC: Akaike Information Criterion
SNP: Single nucleotide polymorphism
NOTCH4: Notch receptor 4
COPZ2: Coatomer protein complex, subunit zeta 2
PDE2A: Phosphodiesterase 2A
SDC1: Syndecan 1
LYPLA2: Lysophospholipase 2
BOK: BCL2 family apoptosis regulator BOK
PRKN: Parkin RBR E3 Ubiquitin Protein Ligase
FEZ1: Fasciculation and elongation protein zeta 1
RASGRP1: RAS guanyl-releasing protein 1
JAKMIP2: Janus kinase and microtubule interacting protein 2
MAPK15: Mitogen-activated protein kinase 15
EMID1: EMI domain containing 1
TP53: Tumor protein 53
TTN: Titin
MUC16: Mucin 16
CIT: Citron Rho-interacting serine/threonine kinase
MEK: Mitogen-activated protein kinase kinase
ERK1/2: Extracellular signal-related kinases 1 and 2
APOA5: Apolipoprotein A5
CHAC1: ChaC glutathione specific gamma-glutamylcyclotransferase 1
EMID1: EMI domain containing 1
GOSR2: Golgi SNAP receptor complex member 2
RGS20: Regulator of G protein signaling 20
RPH3A: Rabphilin 3A
SGCE: Sarcoglycan epsilon
TCP1: t-complex 1
TMEM167A: Transmembrane protein 167A
ZDHHC15: zDHHC palmitoyltransferase 15
