# Supplementary material for: Identification of Molecular Subtypes and Prognostic Features for Triple-Negative Breast Cancer Based on Golgi Apparatus-Related Gene Signature
Source: Oncol Res. 2025 Jul 18;33(8):2013–35. doi: 10.32604/or.2025.061757 (PMC12308265; doi:10.32604/or.2025.061757)
Supplement: Supplementary file 1 [file OncolRes-33-61757-s001.docx]

**Supplementary Figure S1**


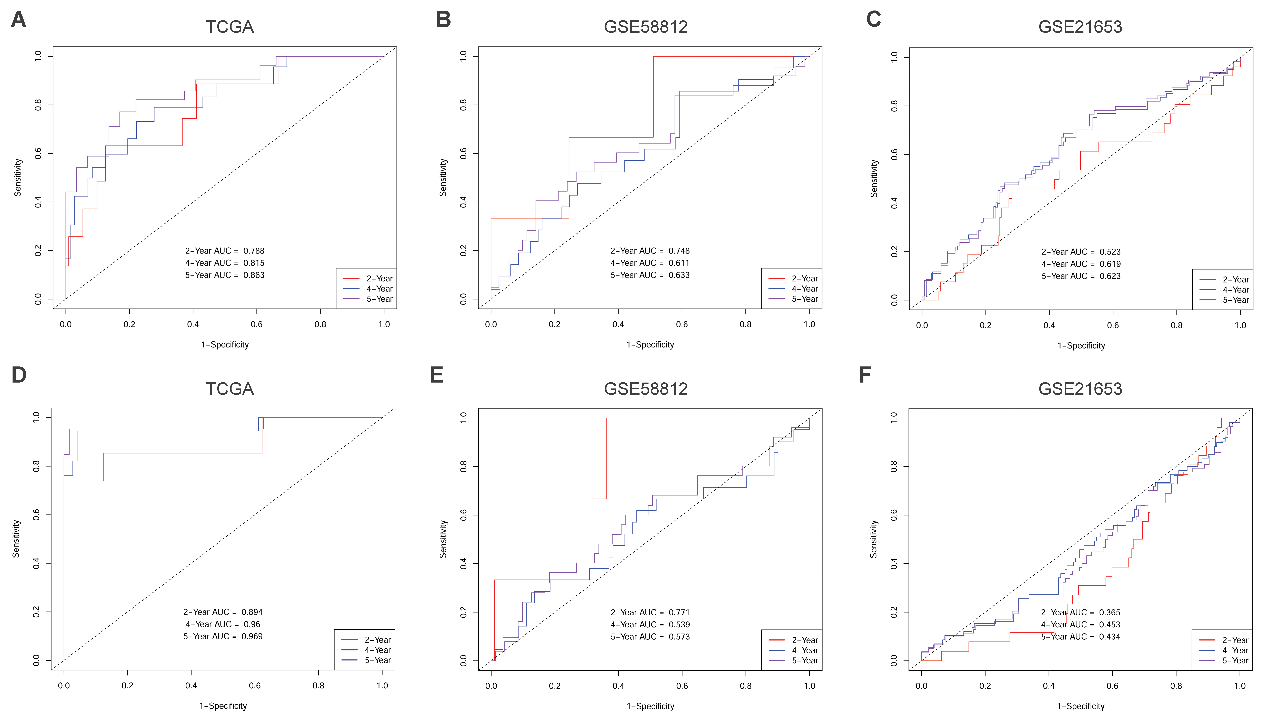


**Supplementary Figure S1** Receiver operating characteristic (ROC) curves showed the performance of this GARGs signature (A-C) and PAM50 gene signature (D-F) in predicting the 2-, 4-, and 5-year OS probabilities based on TCGA, GSE58812, and GSE21653. AUC, area under the curve.
