# Supplementary figures and images for: Identification of Molecular Subtypes and Prognostic Features for Triple-Negative Breast Cancer Based on Golgi Apparatus-Related Gene Signature
Source: Oncol Res. 2025 Jul 18;33(8):2013–35. doi: 10.32604/or.2025.061757 (PMC12308265; doi:10.32604/or.2025.061757)

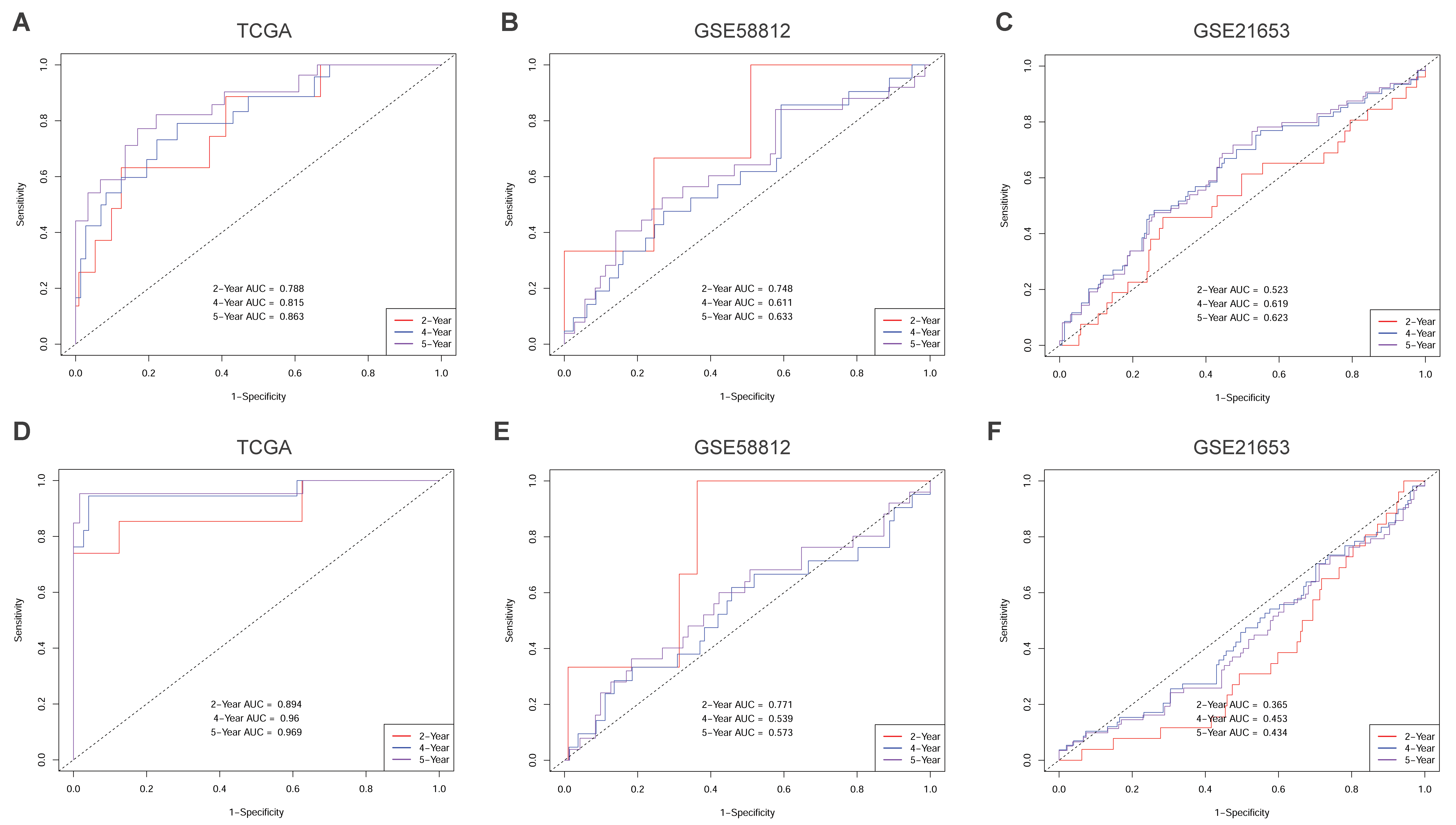

Supplement: Supplementary file 2 [file OncolRes-33-61757-s002.tif]
